# Supplementary material for: Strength of T cell signaling regulates HIV-1 replication and establishment of latency
Source: PLoS Pathog. 2019 May 22;15(5):e1007802. doi: 10.1371/journal.ppat.1007802 (PMC6548398; doi:10.1371/journal.ppat.1007802)
Supplement: S1 File — (PDF) [file ppat.1007802.s001.pdf]

Supplemental materials and methods

Nested *Alu*-PCR

Table 1. Oligos used for *Alu*-PCR.

**Oligos Used for *Alu*-PCR**			
Reaction	Gene / Target	Primer Direction	Primer Sequence
1st Step	*Alu*	Forward	5’-CGTCGCCAGTCAAGTAAC-3′
	luc	Reverse	5’-CTGTAATCCCAGCAGTTTGGGAGGC-3′
2nd Step	3’ R/U5	Forward	5′-GCCTCAATAAAGCTTGCCTTGA-3′
		Reverse	5′-TCCACACTGACTAAAAGGGTCTGA-3′
		Probe	5′-FAM-CCAGAGTCACACAACAGACG-TAMRA-3′

See also Materials and Methods: Nested *Alu*-PCR in the main text.

RT-PCR

Table 2. Oligos used for RT-PCR.

**Oligos Used for RT-PCR**		
Gene / Target	Primer Direction	Primer Sequence
tat	Forward	5’-GGAGCCAGTAGATCCTAGAC-3′
	Reverse	5’-CTTGGCAATGAAAGCAACAC-3′
ACTB	Forward	5’-TGGGACGACATGGAGAA-3′
	Reverse	5′-GGGTGTTGAAGGTCTCAAA-3’

See also Materials and Methods: RT-PCR in the main text.

Chromatin immunoprecipitation

Briefly, cells were washed in PBS and fixed in a final concentration of 1% formaldehyde in methanol. Crosslinking was quenched by addition of glycine to a final concentration of 240mM. Cells were then washed, centrifuged, and flash frozen in liquid nitrogen. Pellets were lysed in Farnham Lysis Buffer (5mM PIPES pH 8.0, 85mM KCl, 0.5% NP-40) with addition of Halt Protease and Phosphatase Inhibitor Single-Use Cocktail from Thermo Fisher before centrifugation to obtain nuclei fraction. Nuclei were lysed in RIPA buffer prior to sonication in Bioruptor Pico for 15 m with alternating 30 s cycles. Samples were centrifuged to remove debris and pre-cleared with addition of 50% protein A sepharose bead for 30 m at 4°C. Beads were then pelleted and supernatants were split into 100μL portions as input DNA and multiple 300μL portions for sample analysis. Samples were incubated with antibodies overnight at 4°C. Antibody-bound proteins and cross-linked DNA were isolated by addition of 50% protein A sepharose beads for 2 h at 4°C prior to centrifugation. Immunoprecipitates were then washed with low salt (0.1% SDS, 1% Triton X-100, 2mM EDTA, 20mM Tris-HCl pH 8.0, 150mM NaCl), high salt (0.1% SDS, 1% Triton X-100, 2mM EDTA, 20mM Tris-HCl pH 8.0, 500mM NaCl), lithium wash (0.25M LiCl, 1% NP-40, 1% sodium deoxycholate, 1mM EDTA, 10mM Tris-HCl), and Tris-EDTA buffers (10mM Tris, 1mM EDTA) before use of elution buffer (1% SDS, 0.1M $NaHCO_3$). Cross-linking was reversed with addition of 5M NaCl overnight at 65°C to both samples and input DNA before addition of proteinase K to isolate DNA. Sample DNA was purified using ChIP DNA Clean & Concentrator Kit (Zymo Research). See also Materials and Methods: Chromatin Immunoprecipitation in the main text. All primers listed in Table 3.

Table 3. Oligos used for Chromatin Immunoprecipitation Assays.

**Oligos Used for Chromatin Immunoprecipitation Assays**		
Gene / Target	Primer Direction	Primer Sequence
TSS* (+30)	Forward	5’-CTGGGAGCTCTCTGGCTAACTA-3′
TSS* (+239)	Reverse	5’-AGATCTCCTCTGGCTTTAC-3′
tat (+5379)	Forward	5’-GGAGCCAGTAGATCCTAGAC-3′
tat (+5482)	Reverse	5’-CTTGGCAATGAAAGCAACAC-3′

* HIV-1 Transcriptional Start Site
