# Supplementary figures and images for: Strength of T cell signaling regulates HIV-1 replication and establishment of latency
Source: PLoS Pathog. 2019 May 22;15(5):e1007802. doi: 10.1371/journal.ppat.1007802 (PMC6548398; doi:10.1371/journal.ppat.1007802)

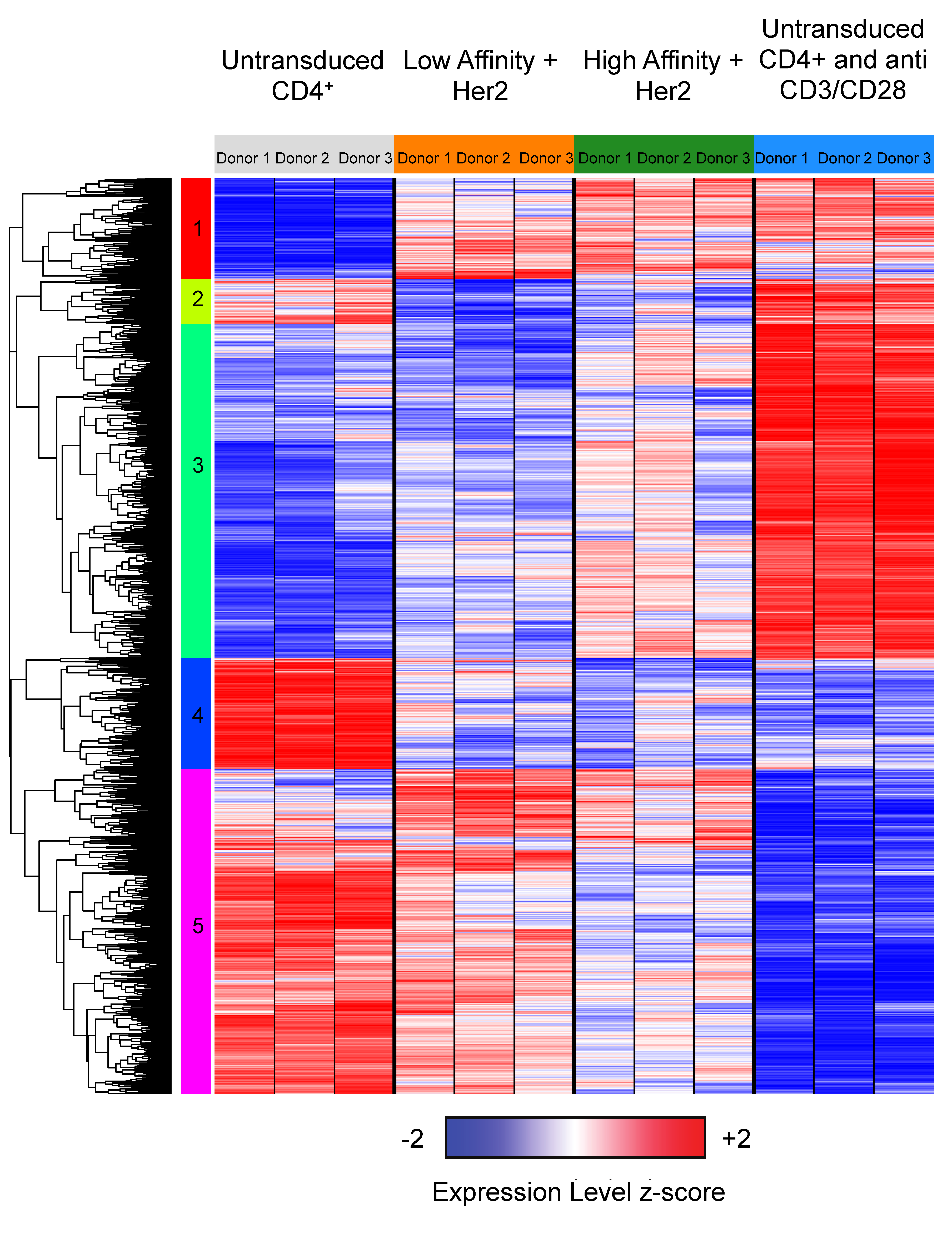

Supplement: S1 Fig — CD4+ T cells isolated from healthy human donors were transduced with low affinity or high affinity CARs and then allowed to return to a resting state as measured by CD69 expression. Cells were then stimulated through the receptor. As controls, untransduced cells were cultured with or without antibodies to CD3 and CD28. RNA was isolated 24 h later and converted to cDNA before being analyzed on a Human Clariom S array. Gene expression levels for cells stimulated through both CARs and TCR-stimulated CD4+ T cells were compared to unstimulated cells. All genes with a one-way ANOVA FDR-corrected q value of < 0.01 were plotted and clustered arbitrarily according to expression profiles. Data is presented as a heatmap based on RNA log2 expression and represents three independent donors. Donors 1 and 3 are female, while Donor 2 is male. Determination of donor gender is described in greater detail in Materials and Methods. See also S1 Appendix “List of genes whose expression is significantly altered upon TCR stimulation.” (TIF) [file ppat.1007802.s002.tif]

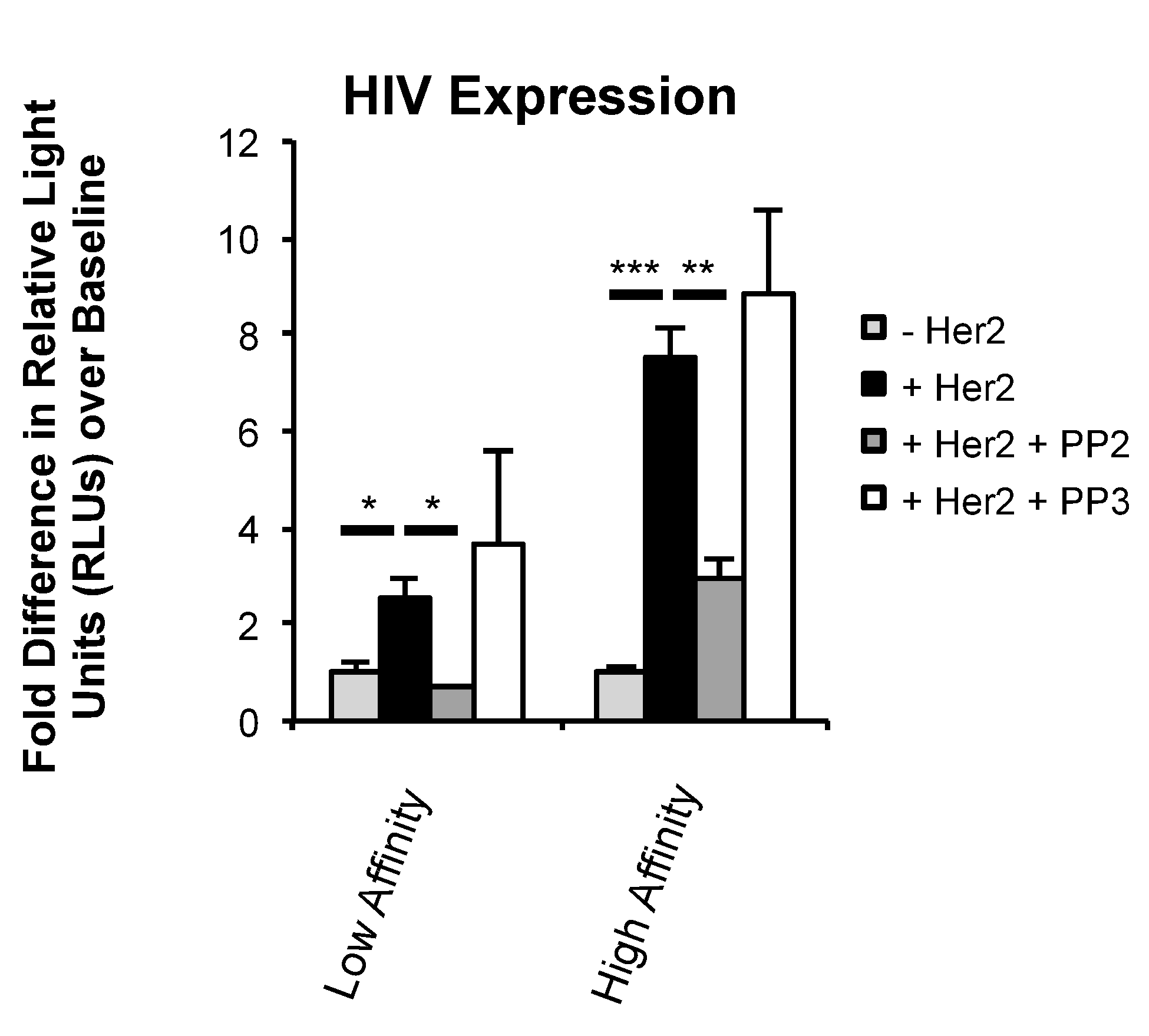

Supplement: S2 Fig — CAR+ Jurkat T cells were stimulated with or without Her2 in the absence or presence of 10 μM PP2 or PP3 at the time of HIV-1 infection with single-round VSV-G pseudotyped NL4-3.Luc. 24 h post infection, cells were lysed to measure luciferase. Data are presented as fold difference in RLUs over unstimulated cells for each CAR+ population. S2 Fig was performed in triplicate and is representative of five independent experiments. Data are presented as mean ± standard deviation. Statistical analysis performed using unpaired Student’s t test and compared to Her2-stimulated conditions. *p<0.01, **p<0.001, ***p<0.0001. (TIF) [file ppat.1007802.s003.tif]

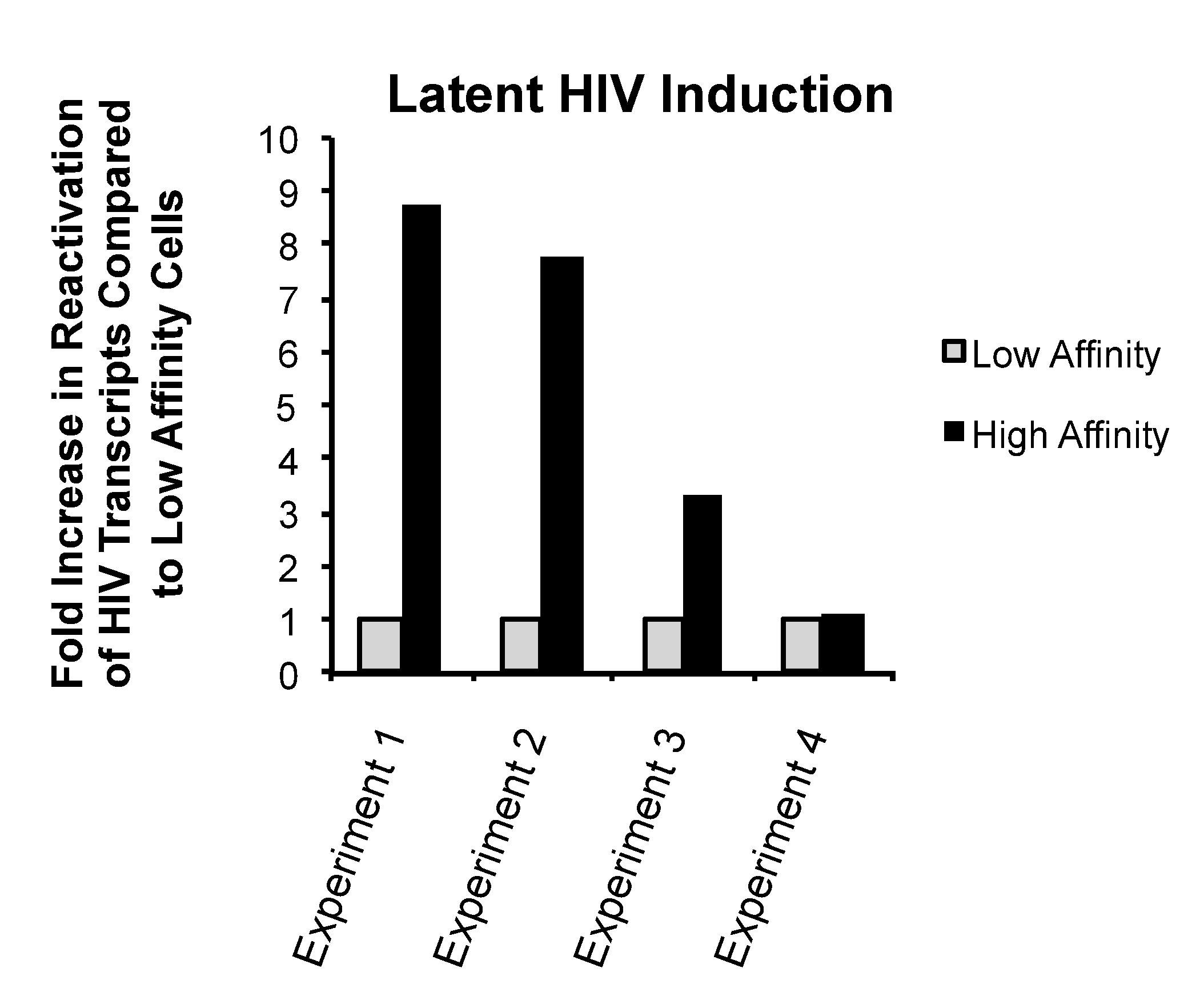

Supplement: S3 Fig — Latently infected cells were restimulated with PMA and ionomycin. HIV-1 expression was monitored by measuring Tat RNA by qRT-PCR. For each assay, the fold difference in HIV-1 transcripts over corresponding non-reactivated controls were normalized to the induction observed in the reactivated low-affinity condition. In this way, multiple assays could be directly compared in spite of differences in the level of induction measured due to donor-to-donor variability. The average fold increase in the level of induction observed in the high affinity population across all experiments is 5.23. Data in S3 Fig are presented as mean of 2–4 replicates and are derived from 3 different donors. (TIF) [file ppat.1007802.s004.tif]
